# Crafting a powerful shield: Unveiling the potent anti-oxidant magic of ex-situ nanostructured Ag/WO_3_ composite

**DOI:** 10.1016/j.heliyon.2024.e25591

**Published:** 2024-02-04

**Authors:** Maria Khizar, Wajeehah Shahid, Samiah Shahid, M.I. Khan, Nawal Ansar, Sana Ullah, Aftab Farrukh, Jeong Ryeol Choi

**Affiliations:** a Department of Physics, The University of Lahore, Lahore 54000, Pakistan; b Institute of Molecular and Biology & Biotechnology, The University of Lahore, Lahore 54000, Pakistan; c Department of Physics, PMAS-Arid Agriculture University, Rawalpindi, 46300, Pakistan; d Department of Nanoengineering, Kyonggi University, Suwon 16227, South Korea

**Keywords:** Tungsten oxide, Silver, Hydrothermal synthesis, Nano composite, DPPH, Anti-oxidant

## Abstract

The current study focuses the nanocomposites of Ag/WO_3_ was synthesized via hydrothermal method and extract of Aloe-vera gel was used. Various characterization techniques were used for the analysis of Ag/WO_3_ nanocomposites which includes SEM (scanning electron microscope), EDX (Energy dispersive spectroscopy), XRD (X-ray diffraction), FTIR (Fourier transform infrared), UV (ultraviolet–visible-spectroscopy) to tell about elemental composition, shape and crystalline structure, band gap, functional group. The presence of composition of elements O, W, Ag in Ag/WO_3_ nanocomposites was confirmed through EDX spectrum. The hexagonal crystal structure and the border peaks in Ag/WO_3_ nanocomposites were examined through XRD spectra. The Anti-oxidant activity was synthesized by using (DPPH) free Radical in Ag/WO_3_ nanocomposites. The outcomes of present study exhibited an excellent anti-oxidant activity and also indicated the reduction of stabilized free radical DPPH analysis using Aloe vera extract. The result revealed that the anti-oxidant activity of Ag/WO_3_ nanocomposites is essential for biomedical application and various industries.

## 1 Introduction

Nanocomposites incorporated together with two or more phases or components with varying physical and chemical properties, which has a unique feature from its constituent elements. Materials reinforced the nano structures referred as a nanocomposite [1]. Metal -organic -frame work (MOF) based nanocomposites are utilized in wide range of application including as biomedical imaging, enzymes activity, biosensing, energy gas detectors, hydrogen storage mechanism and it extensively employed innumerous different industries such as treatment of waste water, optical and electronic equipment's, nanotechnology devices, protective coating, electroconductive frameworks supercapacitors, photo voltaic cells and development of renewable energy [2]. WO_3_ is an n-type semiconductor every additional metal contained vacancy of oxygen is essentially creates the stiochmetric action. The amount of charge carrier density at the surface is diminished in the instance of n-type metal oxide, consequently, band gap is created when the electrons initiating from the donors through conduction band (CB) [3]. Although WO_3_ is capable of triggering a wide range of process and its potential effect in antioxidant activity is currently examined. WO_3_ is basically being used for gas detection [4] for the ultra-violet (UV) adsorption. WO_3_ has an indirect band gap which is smaller than 3.0eV [5]. WO_3_ has been utilized in variety of unique physical characteristics which involves electro-chromium [6], thermal energy conservation [7], as well as light adsorption [8]. Moreover, it is environmental friendly thermally resistance, chemical undetectable, and entirely suitable to use in everywhere. Tungsten (WO_3_) has a one of most promising characteristics and wide range of applicant in different areas which includes gas sensor [9,10]capacitors, electrical equipment's lithium ion batteries [11], dye synthesized photo-voltaic cell [12,13], photo-catalysis [14,15], electrochromic gadget [16,17], adsorption material [18], bio-catalysts and drug delivery carrier [19]. In biological field WO_3_ NPs are exhibit promises in initiating particular steps that may scavenger the free Redical as well as enhance the antioxidant activity. WO_3_ have numerous bio-medical applications in particular areas of bio-imaging, antibacterial cancer therapy, biological detection, as well as bacterial protection.

Due to distinctive properties, the Silver NPs in addition to other type of nano-particles; have been attracted enormous attention [20]. Silver NPs may contain some antioxidant characteristics because of these Silver NPs have distinct properties as well as an antioxidant effects which have been studies for a wide range of therapeutic uses. A lot of research has been investigated on Silver NPs owing to its biological activity which involves anti-oxidant [21], anti-fungal, anti-bacterial properties [22]. Silver NPs are being examined for their capcity to lower the level of oxidative stress in cells as well as scavange the harmful free radicals. As a result the Ag NPs nanoparticles are currently used in wide range of applications in various fields which includes a delivery of medication, fabrication of nano devices [23], therpecutatic [24]and bio-medical fields [25]. The implementation of AgNPs is essential and exciting technology in a drug delivery [26]. Furthermore, the plant based compound has ability to accumulate in to the nanoparticles edges [27,28]. Several plant extract are being used to produce the different kinds of nanoparticles which includes as Au, ZnO [29], Ag [30] and several others [31,32] Succulent plant of aloe-vera which is member of the lily (Liliaceae species) had a broad range of potetntional therpecutatic properties. According to recent report Aloe-vera gel composed of approximately 75 micro-nutrients, minerals as well as 200 substances that are biologically active such as nutrients, digestive enzymes, glucose, callouses, amino acid, saponins [33]. The previous investigation demonstrates the aloe-vera gel natural extract had a strong antioxidant ability in both vitro or in vivo [34]. The anti-oxidant capability of AgNPs fabricated from aloe vera-gel extract have not been examined [35].

The main objective of the current study to investigate the in vitro antioxidant ability of Ag/WO_3_ nanocomposites through the water-based gel of aloe vera extract. Furthermore, no previous research was examined the fabrication of AgNPs, WO_3_ NPs and Ag/WO_3_ nanocomposites to evaluate the anti-oxidant potential applications. In current study, synthesis of WO_3_ NPs, Ag NPs and Ag/WO_3_ nanocomposites was carried out via aloe vera gel extract which act as a stabilizing, reducing and capping agents and their biological activities have been investigated to study anti-oxidant potential applications.

## 2 Material and methods

All the chemicals were used in this research were of analytical grade, acquired from sigma Aldrich Company. They were all utilized without additional purification. The precursor of silver nitrate and sodium tungstate dehydrate was used for the fabrication of WO_3_ and Ag nanocomposites.

### 2.1 Synthesis of aloe-vera leaf extract

**Fig. .1:**
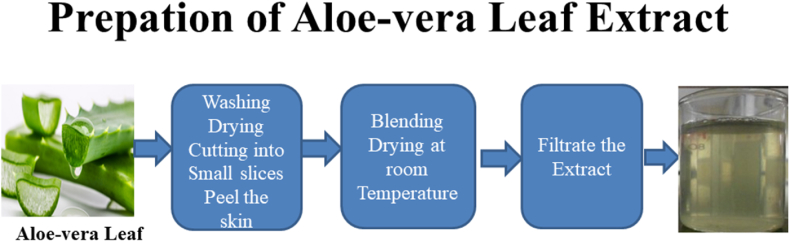
Schematic diagram of prepation of Aloe-vera Extract.

Fresh aloe Vera leafs were collected from plant for the prepation of aloe Vera extract. For this purpose initially take aloe vera leaf and wash the leaf with distilled water and ethanol completely. After we dried them leaf at room temperature for 10 min and sliced in to slight bits then peel the skin of the aloe vera leaf. 400 g Aloe vera gel was taken and blended the aloe vera extract with equivalent amount of distilled water. Then subsequently the extract solution was placed in oven for 15 min at 80 °C. After wards the prepared extract solution was filtered through the funnel. In this process filter paper is used. So, finally aloe vera extract solution was synthesized. The following Fig. 1 depicts the schematic diagram of prepation of Aloe-vera leaf Extract.

### 2.2 Synthesis of WO_3_ nanoparticles

**Fig. .2:**
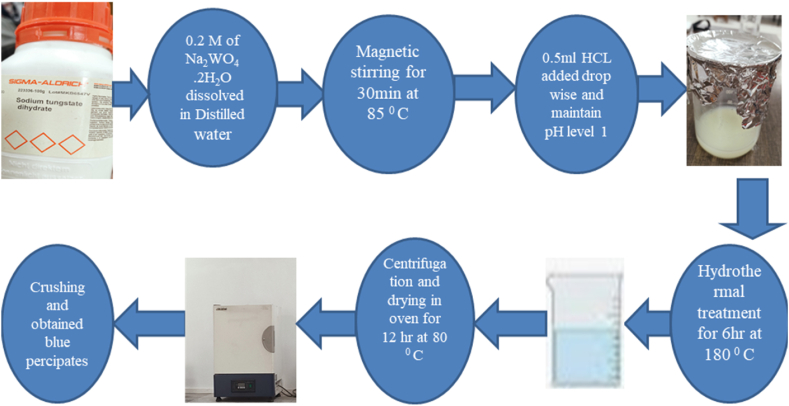
Schematic diagram of synthesis of WO_3_.

The hydrothermal process was utilized for the synthesis of Tungsten-trioxide (WO_3_) NPs. For the fabrication of WO_3_ NPs 0.2 M of sodium tungstate dehydrates (Na_2_WO_4_.2H_2_O) which was liquefied in distilled water 40 ml to retain the pH level of ∼8. The 0.5 M Hydrochloric Acid (HCL) was added drop by drop at 50 °C. The obtained solution pH range was changed to ∼1 after during vigorous stirring for 15min.Before the hydrothermal process sonicated the above mixture for 2 h. The above solution was transformed in to 50 ml Teflon-stain less steel auto-clave and retained in an oven at 180 °C for 6 h. After this allow the solution for refrigerating the liquid at ambient temperature and then placed the desired solution in to centrifugation tubes. To eliminate the impurities, the precipitated nanoparticles were washed with two or thrice times using ethanol and distilled-water. Put the above mixture in oven and dried it at 80 °C for 12 h. Finally, the blue colour precipitate's was achieved after grinding. Fig. 2 given below show the schematic diagram of synthesis of WO_3_

### 2.3 Synthesis of Ag nanoparticles

**Fig. 3:**
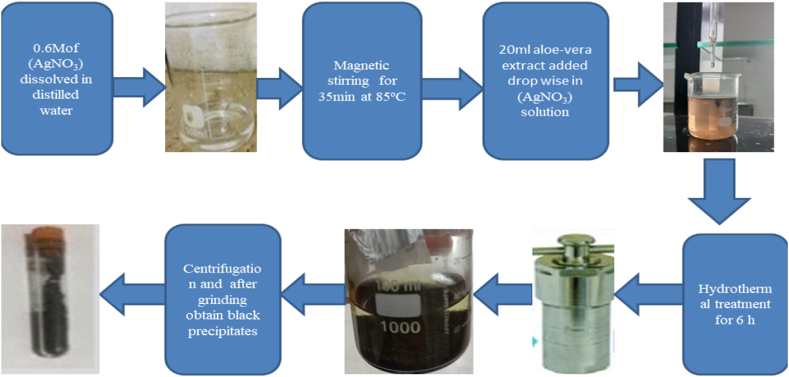
Schematic diagram of synthesis of Ag nanoparticles.

20 ml aloe vera leaves extract was added drop-by-drop to 0.6 M and 20 ml aqueous mixture of Silver nitrate (AgNO_3_) under vigorous stirring at 85 °C for 35min. The above solution was kept on sonication for 2 h. Then the prepared mixture was transformed in to 50 ml Teflon-stain less steel auto-clave and maintained the temperature 150 °C in an oven for 6 h. The resultant solution was converted into blackish paste after hydrothermal process signifying that the silver nitrate was transformed into Ag NPs with the addition of aloe vera leaves as a reducing agent. The obtained mixture was centrifuged for 10 min at 4500 rpm and the final product was dried in an oven at 80 °C. Furthermore, the obtained blackish mixture was converted into dark blackish precipitations which indicate the Ag NPs. The following Fig. 3 represents the schematic diagram of synthesis of Ag nanoparticles.

### 2.4 Ex-situ synthesis of Ag/WO_3_ nano-composites

**Fig. 4:**
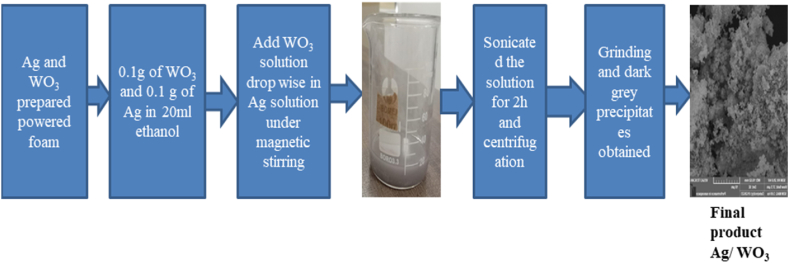
Schematic diagram of synthesis of Ag/WO_3_ Nanocomposites.

Ag/WO_3_ nanocomposites have been fabricated by utilizing an Ex-situ synthesis method. WO_3_ and Ag nano-particles that have already been prepared were employed in this process. First of all 0.2 g of WO_3_ and 0.2 g of Ag were mixed to 20 ml of ethanol to make nanocomposites by the same molar ratio of 1: 1 and stirred continuously for 30 min. Add the drops of Ag solution in to WO_3_ solution through magnetic stirring for 1 h. The resulting mixture is further sonicated for 1 h. The above solution was centrifuged to gather particles and washed several times with ethanol as well as distilled water. After that the final product was dried in an oven for 6 h at 80 °C and dark grey precipitations were obtained. Fig. 4 given below describe the schematic diagram of synthesis of Ag/WO_3_ Nanocomposites.

## 3 Results and discussion

### 3.1 XRD analysis

**Fig. 5:**
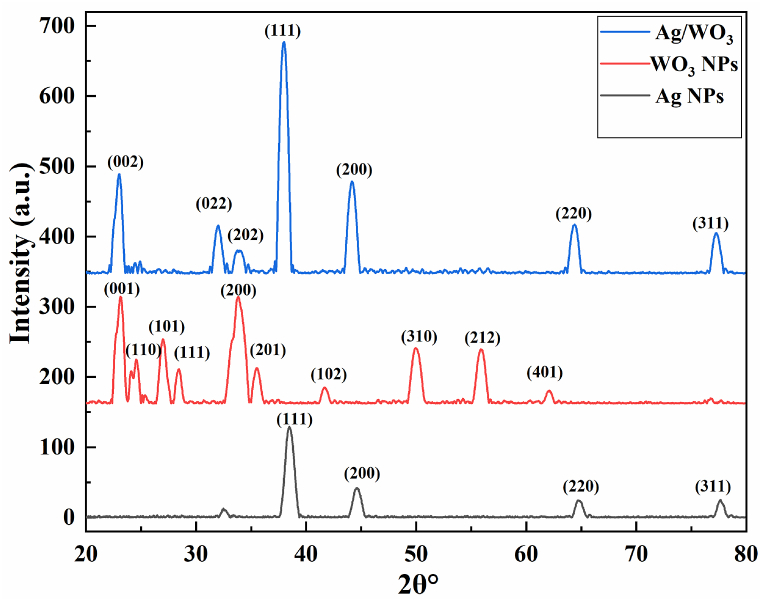
XRD pattern of Ag/WO_3_ nanocomposites.

The crystallographic structures of prepared sample of Ag/WO_3_ nanocomposites were investigated via XRD analysis. The technique of XRD was accomplished via Cu Ka frequency of (1.540 A^0^) illustrated in Fig. 5. In XRD the diffraction angles are in the range of 20-80^0^ [36]. Four distinctive sharp peaks were observed in XRD spectra of Ag NPs and these peaks were located at different angles at 38.5^0^,44.7^0^,65.24^0^,78.7^0^ and their parallel hkl values are (111), (200),(220),(311) which confirms the formation cubic phase of Ag NP's respectively [37]. In XRD the recorded data match with (JCPD Card no 04–0783) [38]. It is evident from the XRD analysis that WO_3_ NPs major peaks at 22.8^0^, 24.6^0^, 28.3^0^, 33.78^0^, 34.6^0^ 41.6^0^, 49.8^0^, 55.8^0^,61.8^0^,76.4^0^. The corresponding miller indices planes marked in a Fig. 5. (001), (110), (101),(111),(200), (201), (102), (310), (212), (401) which confirms the tetragonal phase of WO_3_ nano structures and all the peaks match with (JCPD card no 05–0388) of WO_3_. Various diffraction peaks were located in Ag/WO_3_ nanocomposites of XRD spectra at 22.9^0^, 32.1^0^, 33.9^0^, 38.5^0^, 44.7^0^, 65.2^0^ and 78.7^0^. The different peaks of diffraction and these indices (002), (022), (202), (111), (200), (220), (311) labeled on XRD pattern three peaks (002,(022),(202) shows the WO_3_ peaks at diffraction angles 22.9^0^, 32.1^0^, 33.9^0^, and correspondingly matched with (JCPD card no 33–1195 and 83–0950) as well as four peaks (111),(200),(220),(311) and their different angles at 38.5^0^,44.7^0^,65.24^0^,78.7^0^ exhibits the Ag NP, and its match with JCPD card no 01-087-0719 which confirms the formation cubic phase of Ag NPs [39,40] Sharp peaks were obtained in XRD spectrum which confirmed the hexagonal phase of preparation of Ag/WO_3_ nanocomposites. XRD results showed that there no impurity peaks was originated from Ag/WO_3_ nano composites [41]. The observed pattern demonstrated that in WO_3_ broad peaks was examined as compared to Ag. In XRD pattern broadening peak was influenced by the size of crystallites and the peak is broader due to small crystalline size. All the shapes of distinctive peaks of pure Ag/WO_3_ nanocomposites are observed in XRD.

### 3.2 FTIR analysis

**Fig. 6:**
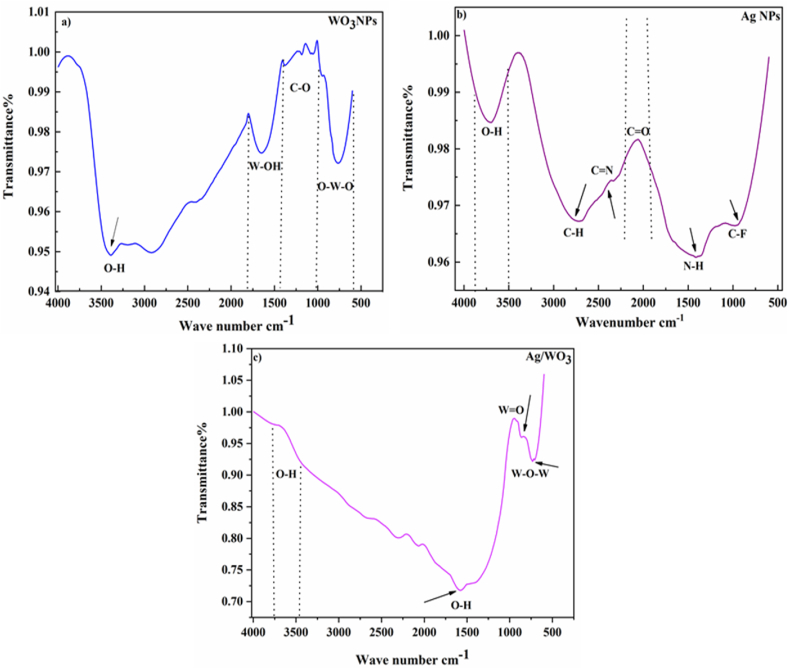
[(a–c)]. Fourier Transmission Spectrum of Ag/WO_3_ nanocomposites.

The chemical properties of substances are identified through Fourier transform spectroscopy (FTIR). This Fig. 6[(a)] illustrated the FTIR spectrum which ranges from 4000 to 500 cm^−1^ of WO_3_ nano materials [42]. The purity of WO_3_ nano material identified to absorption spectra at 3387.07,1640.99,1388.48,948.21,765.51 cm^−1^ produced to several functional group (O–H) hydroxyl,(W–OH), tungsten hydroxyl group,(C–O),carbonyl, (O–W–O). A border peak was observed at 3387.07 cm^−1^ and attributed with (O–H) group of bending vibration [43]. This stretching band is due to water molecules which exist in WO_3_ nano structures. The absorption spectra in the region of 1640.99 cm^−1^ and 1388.48 cm^−1^ that are inter linked with the tungsten group of hydroxyl. A very small and sharp band was identified at 948.21 cm^−1^ that are co related with (C–O) carbonyl group which is slightly moved that is near about to 1000 cm^−1^. The stretching vibration of O–W–O located in the region of 765.51 cm^−1^ [44]. The FTIR spectra of Ag nano material shows the different peaks and various functional groups as represented in Fig. 6[(b)]. The purity of nano structures demonstrated the absorption bands at 3713.71, 2712.38, 2358.52, 1407.21,1014.24 cm^−1^ generated to various functional groups O–H (Hydroxyl), C=N nitrile groups,C=O carbonyl groups,N–H amine –I,C–F (Fluorides) [45]. The band spectrum at 3713 cm^−1^ showed that a border peak the can be interlinked with (O–H) alcoholic group of asymmetric vibrations. The spectrum at 2712.38 cm^−1^ that are related to (C–H) alkane group. A very small peak was observed at 2358.52 cm^−1^ that can be associated to C=N (nitrile) group as a bending vibration. The band spectra at 1735.98 cm^−1^ which was interlinked with (C=O) carbonyl group and this group is made up of carbon atom with the (=) bond of Oxygen atom [46]. The spectrum present at the center at 1407.31 cm^−1^ which is correlated with (N–H) Amine-I group. The vibrational group of (C–F) fluoride compounds is located in the region of 1014.24 cm^−1^ [47]. These FTIR absorption bands confirmed the Ag NPs which can be act as stabilizing and reducing agent. Furthermore, the syntheses of (Ag/WO_3_) silver and tungsten oxide nanocomposites were displayed in Fig. 6[(c)]. The absorption spectra at 3687, 1614, 910, 710 cm^−1^ ascribed to bending vibration of (O–H), W=O, W–O–W [48]. Broader band was identified at 3687 and 1614 cm^−1^ that co-relates with the stretching modes of (O–H) hydroxyl group [49]. Asymmetric vibration of (O–H) is due to ethanol use in Ag/WO_3_ nano-composites. Smaller peak was located at 910 cm^−1^ attributed to the (W–O–W) [50] bending vibration and it overlap with (W=O) at 1000 cm^−1^ [51]. Stronger peak observed at 703 cm^−1^ that are interlinked with (W–O–W) to bending modes [52]. This FTIR confirms the formation of Ag/WO_3_ nano composites.

### 3.3 UV- spectroscopy

UV–visible spectrometer was utilized to measure the optical absorption spectrum of chemically synthesized sample of the WO_3,_ Ag as well as Ag/WO_3_ nanocomposites and its effective wave length vary round about 200–800 nm [53]. The optical band gap was measured using Tauc formula of the prepared samples.

(1) $$\alpha hv=A\;{\left(hv-\;E_{g}\right)}^{n}$$

**Fig. 7:**
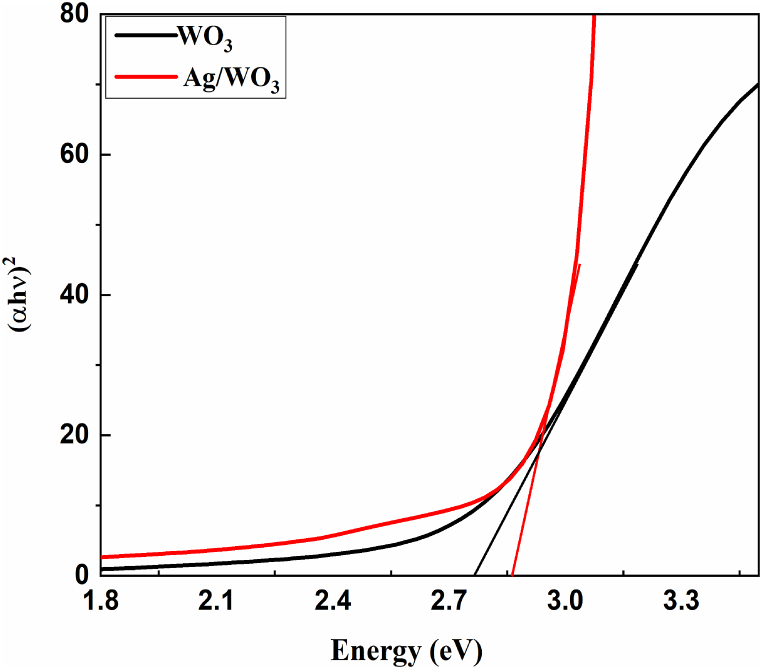
UV–Visible Spectroscopy of Ag/WO_3_ nanocomposites.

The above equation (1) υ basically denotes the frequency, “E_g_” is energy band gap, α and represents the absorption parameter, h Symbolize the plank constant, n is basically absolute number that various kinds of transition. All the prepared samples exhibits the direct band gap values of 2.7eV [54], 2.80eV of WO_3_, as well as Ag/WO_3_ composites depicted in Fig. 7. When Ag is incorporated into WO_3_ it expanded the band gap of energy in Ag/WO_3_ nanocomposites because the electrons and holes at nano scale are confined due to the quantum confinement effect, the variation in the energy among the filled and empty region broaden consequently which leads to widening the band gap of semi conductivity.

### 3.4 SEM analysis

**Fig. .8[(a–f)]:**
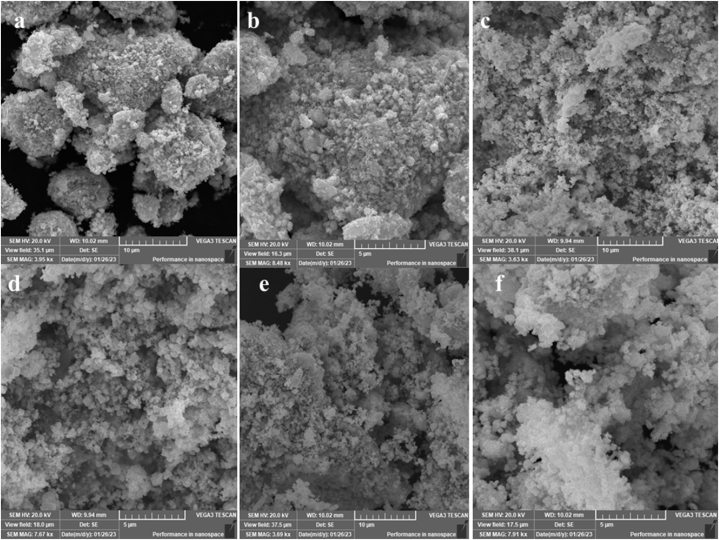
SEM micrograph of Ag/WO_3_ nanocomposites.

Surface morphology was confirmed by SEM analysis of prepared 10 μ m and 5 μ m of WO_3_, Ag, Ag/WO_3_ nano composites. The SEM analysis of prepared WO_3_ nanostructures was represented in Fig. 8[(a-b)]. The SEM images of WO_3_ nanostructures which exhibit the creation of nano materials [55]. SEM clearly indicates the structural properties including a structure and its shape. The morphology of nano particles always displays in micro scale. In SEM Fig. 8[(a)] which is 10 μm are relatively larger in size as compared to Fig. 8[(b)] 5 μm. This SEM analysis clearly shows the shape of WO_3_ nanostructure which is spherical agglomeration and homogenously dispersive across the region [56]. The WO_3_ NPs are arbitrary placed in various directions. The SEM morphology reveals that amalgamation of nano particles and the development of nano structures. Furthermore, SEM analysis of silver nanostructures was displayed in Fig. 8[(c-d)] were examined. The Ag NPs was produced by using the aloe-vera extract which can be used as oxidizing agents. The Ag NPs indicated that the morphology is in spherical shape [57]. The assembled cluster were observed in SEM images to be scattered around the region with an enormous amount of arbitrary empty spaces [58]. The SEM images revealed that the Ag NPs were equally disturbed over the region. The SEM micrographs confirmed the synthesis of Ag NPs. Fig. 8[(e-f)] displays the formation of SEM micrograph of metal oxide and their composites. The morphology of Ag/WO_3_ reveals the spherical and irregular shapes [59]. The composite which is prepared through aloe-vera solution exhibited spherical non uniform and irregular foam of microstructure. Particles seemed to be distributed arbitrary in variety of cluster. The morphological and structural changes analyzes through SEM technique. Dark areas show the existence of Ag and white portion show the existence of WO_3_ which is clearly show in SEM micrograph [60]. SEM analysis confirmed the formation of Ag/WO_3_ nanocomposites.

### 3.5 EDX analysis

**Fig. 9:**
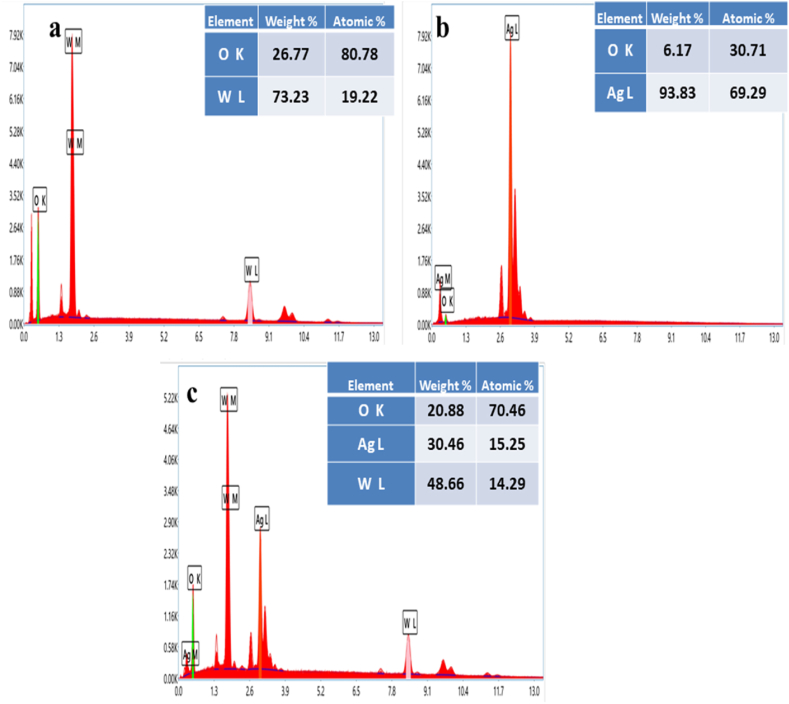
[(a–c)]. EDX spectra of Ag/WO_3_ nanocomposites.

EDX analysis is one of the most effective methods to determine the synthesis of prepared WO_3_, Ag, Ag/WO_3_ nano composites. The chemical composition of the WO_3_ nano structures were analyzed through EDX. Fig. 9[(a)] depicts the elemental composition of WO_3_ using EDX analysis. The observed spectra demonstrating the W and O as the primary elements in synthesis of WO_3_ nanomaterial. The weight percentage W and O 73.23 % age 26.77 % and atomic percentage of O, W were 80.78 % and 19.22 %. The atomic percentage of Oxygen is two times higher than Tungsten (W) in WO_3_. The observed peaks of Tungsten (W) and Oxygen (O) depicted on the EDX figure at the various energies 8.43 keV, 2.35kev and 0.51kev, respectively [61]. The result clearly shows that there is no impurity exists in the WO_3_ material due to absence of additional components [62]. The nano structure of WO_3_ confirms via EDX spectra. Fig. 9[(b)] illustrates the given spectra of Ag NPs using EDX analysis. Silver is main component of element parallel to Oxygen in synthesis of Ag NPs. EDX figure revealed that two peaks were plotted in a graph one is higher and other is weaker. High peak exhibited the silver nano structure at 3kev and the less peak indicated the oxygen which might have emerged from the biomaterials that are attached to silver interface demonstrating the silver nanostructures were oxidized to elemental silver. In Ag NPs nano structures the atomic percentage of Ag and O 69.29 % , 30.71 % and weight percentage of Ag and O were 93.83 % 6.17% [63]. EDX spectra confirm the preparation of silver nanostructures because high peak at 3kev was appeared in the silver region. EDX plot of Ag/WO_3_ nano composites are illustrated in Fig. 9[(c)] which demonstrating the existence of three basic elements such as W, O, Ag. The observed spectrum showed the existence of three different peaks of Tungsten (W), Oxygen (O) and Silver (Ag). The atomic percentage of W, O, Ag, 14.29 %, 70.46 %, 15.25 % and weight percentage of Ag, W, O 30.46 % 48.66 %, 20.88 %. Largest peak was seemed in tungsten element as compared to oxygen and silver. The graph demonstrated that nano composites of Ag/WO_3_ no impurities exist due to non-existence of extra elements. The Ag/WO_3_ nano composites results confirms through the EDX.

### 3.6 Anti-oxidant activity

The free Radical associated compound (DPPH) 2,2 diphenyl −1, picryl hydrazyl method which is commonly employed to check the substance capability to server as free radical removal and to examine the effectiveness of the anti-oxidant [64,65]. (DPPH) is a very quick, simple and cost effective technique to determine the antioxidant ability of material. Free radical removal activity of various Aloe-vera plants extract were examined via (DPPH) method [66]. In a brief manner the Ag/WO_3_ nanocomposites were intermixed in distilled water after being the effect of sonication for 30min. The precise concentration of DPPH mixture was prepared in ethanol 0.004 %. 3 mL of DPPH solution were combined to each sample which amounted to around 100 μ L. Only those concentration that are soluble in ethanol was utilized in this whole scenario and the different amounts of those concentration were producing through dilution procedure. The entire mixture was vigorously mixed well and left the prepared mixture for half hour at ambient temperature in dark area. At 517 nm absorption was determined by applying the UV-VIS Spectroscopy. Higher level of activity of free radical was demonstrated through the reaction mixture of low absorption. Ag/WO_3_ nano composites of in vitro studies of anti-oxidant capbitilty was accessed and captured by using the method of (DPPH) 2,2,-diphenyl-1-1 picrylyhydrazyl free Radical elimination technique [67,68]

The given equation (2) below was used to determine the amount of inhibition of free Radical elimination method which is displayed as

Eq (2) $$\text{Free}\;\text{Radical}\;\text{anti}-\text{oxidant}\;\text{activity}=\frac{\left(\;Ac-As\right)}{Ac}\;x100$$

A_c=_indicates the absorbing capacity of the control action.

A_s_ = indicates the absorption capacity of test specimen.

The antioxidant activity was accessed via (DDPH) 2, 2,-diphenyl-1-1 picrylyhydrazyl free Radical elimination technique [69]. The following table [1] represents the Antioxidant activity of Ag/WO_3_ nanocomposites using DPPH free radical (see Table 1).

**Table 1:** Antioxidant activity of Ag/WO_3_ nanocomposites using DPPH free radical.

Sr. Number	Material	Percentage inhibition
1	WO_3_	63.285 ± 0.035
2	Ag	68.82 3± 1.16
3	Ag/WO_3_	70.133 ± 0.720

Values are displayed as mean ± stand deviation if n = 3.

**Fig. 10:**
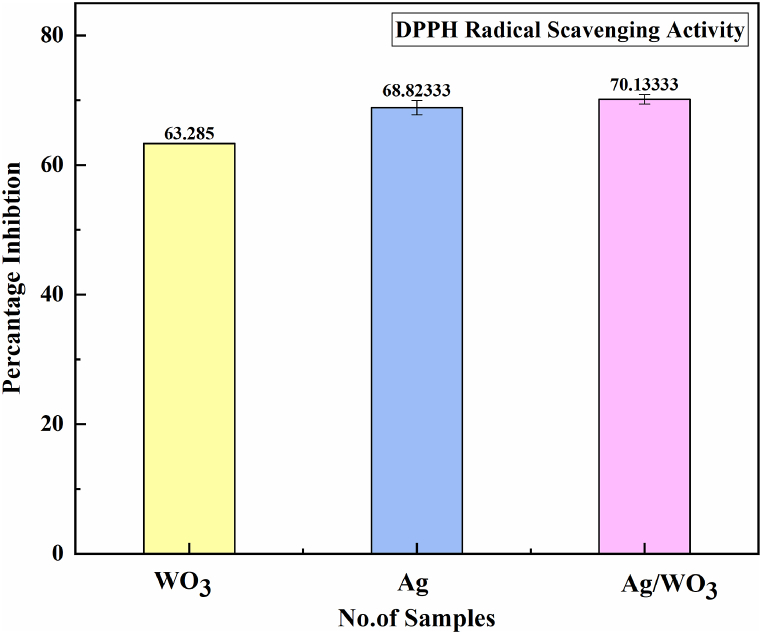
Antioxidant Activity of Ag/WO_3_ nanocomposites.

By evaluating the capability of samples extracts to transfer electrons or donates hydrogen to DPPH, transforming it into its reduced form, the anti-oxidant activity of nanocomposites was analyzed. The percentage of inhibition of (DPPH) free radical was displayed according to their findings. The anti-oxidant ability of Ag/WO_3_ nanocomposites changed according to their concentration. The DPPH inhibitory capacity of samples WO_3_, Ag, Ag/WO_3_ nano composites was calculated to be 63.285 ± 0.035, 68.82 3± 1.16, 70.133 ± 0.720 accordingly. Free Radical anti-oxidant activity of WO_3_, Ag, Ag/WO_3_ nano composites were depicted in Fig. 10. The Ag/WO_3_ NPs exhibited the highest percentage inhibition level which indicates strongest anti-oxidant capacity of all the samples. According to reported literature [70,71] the nanocomposite has high surface-to-volume makes them particular effective anti-oxidants. The present study indicates the anti-oxidant activity of Ag/WO_3_ nanocomposites and no previous literature was found regarding to determination of anti-oxidant of Ag/WO_3_ nanocomposites.

## 4 Conclusion

Ex-situ synthesis of Ag/WO_3_ nanocomposites was successfully prepared via hydrothermal technique and aloe-vera extract was utilized during the preparation. Several distinguish techniques was used which includes EDX,UV–Vis, XRD, FTIR as well as SEM and the material semi-crystanility and hexagonal phase demonstrated with the help of XRD investigation. The identification of absorption bands during the analysis of FTIR spectroscopy verified the existence of various groups such as carbonyl group, C=N nitrile groups, N–H amine hydroxyl group (O–H) enable the stability and the fabrication of Ag/WO_3_ nanocomposites. Granular spherical like shape was originated in Ag/WO_3_ nanocomposites and prove via SEM micrograph. In Ag/WO_3_ nano composites the weight percentage of Ag, W, O 30.46 % 48.66 %, 20.88 % and atomic percentage of W, O, Ag, 14.29 %, 70.46 %, 15.25 % verified via EDX analysis. The optical values of band gap which includes as 2.7ev, 2.80ev of WO_3_, Ag/WO_3_ nanocomposites, respectively, were examined with the help of UV-spectroscopy. In Ag/WO_3_ nano composites showed the greatest antioxidant activity 70.13 % using DPPH assay of scavenging of free Radical. The results outcome demonstrated that Ag/WO_3_ composites was produced and have good antioxidant capacity using plant extract which is potential in biological remediation as well as bio medical application.

## Author Contributions

M.K designed and performed the experiments and wrote the manuscript. W.S proposed, designed, and supervised all of the research work; S.S helped in performing antioxidant activity; M.I.khan provided characterization facilities; N.A and S.U helped with writing and graphing; and A.F helped in reviewing the manuscript; and J.R.C. helped with the final revision and funding. All authors have read and agreed to the published version of the manuscript. First two authors have equal contribution.

## Funding

This study is supported by the 10.13039/501100003725National Research Foundation of Korea (NRF) grant no. NRF-2021R1F1A1062849.

## Data availability statement

Not applicable.

## Additional information

No additional information is available for this paper.

## Declaration of competing interest

The authors declare the following financial interests/personal relationships which may be considered as potential competing interests:Jeong Ryeol Choi reports article publishing charges was provided by School of Electronic Engineering, Kyonggi University, Suwon 16,227, South Korea,. Jeong Ryeol Choi reports a relationship with School of Electronic Engineering, Kyonggi University, Suwon 16,227, South Korea, that includes: employment. If there are other authors, they declare that they have no known competing financial interests or personal relationships that could have appeared to influence the work reported in this paper.

## References

[1] Bendahou A., Kaddami H., Dufresne A.J.E.P.J. (2010). Investigation on the effect of cellulosic nanoparticles’ morphology on the properties of natural rubber based nanocomposites. European Polymer Journal.

[2] Parameswaranpillai J. (2016).

[3] Hariharan V., et al., A Review on Tungsten Oxide (WO3) and Their Derivatives for Sensor Applications, Int. J. Adv. Sci. Eng.,vol. 5, 2019, pp. 1163–1168.

[4] Wang G., et al., Fabrication and characterization of polycrystalline WO3 nanofibers and their application for ammonia sensing, J Phys Chem B,110 (47) (2006) 23777–23782. 10.1021/jp0635819 17125339

[5] Widenkvist E. (2008). Synthesis of nanostructured tungsten oxide thin films. Crystal Growth & Design.

[6] Wang J. (2008). Synthesis, assembly, and electrochromic properties of uniform. crystalline WO3 nanorods,J. Phys. Chem. C,.

[7] Munawar T. (2022). Fabrication of dual Z-scheme TiO2-WO3-CeO2 heterostructured nanocomposite with enhanced photocatalysis, antibacterial, and electrochemical performance.

[8] Upadhyay S.B. (2014). Structural and alcohol response characteristics of Sn-doped WO3 nanosheets.

[9] Li J. (2015). Hydrothermal synthesis of self-assembled hierarchical tungsten oxides hollow spheres and their gas sensing properties.

[10] Wang C. (2015).

[11] Ma L. (2015). Hydrothermal preparation and supercapacitive performance of flower-like WO3 H2O/reduced graphene oxide composite.

[12] Qi H. (2014). Triple-layered nanostructured WO 3 photoanodes with enhanced photocurrent generation and superior stability for photoelectrochemical solar energy conversion.

[13] Prabhu N. (2014). Enhanced photovoltaic performance of WO 3 nanoparticles added dye sensitized solar cells.

[14] Li J. (2013). Microwave-assisted growth of WO 3· 0.33 H 2 O micro/nanostructures with enhanced visible light photocatalytic properties.

[15] Aslam I. (2014). Synthesis of three-dimensional WO 3 octahedra: characterization, optical and efficient photocatalytic properties.

[16] Ou J.Z. (2012). The anodized crystalline WO 3 nanoporous network with enhanced electrochromic properties.

[17] Xiao W. (2013). Na 2 SO 4-assisted synthesis of hexagonal-phase WO 3 nanosheet assemblies with applicable electrochromic and adsorption properties.

[18] Li J. (2013). Formation of WO 3 nanotube-based bundles directed by NaHSO 4 and its application in water treatment.

[19] D'Arienzo M. (2014). Surface interaction of WO 3 nanocrystals with NH 3. Role of the exposed crystal surfaces and porous structure in enhancing the electrical response.

[20] Alharbi N.S. (2022). Green synthesis of silver nanoparticles using medicinal plants: Characterization and application.

[21] Marimuthu S.J.P.R., Santhoshkumar Th, Kirthi A.V., Jayaseelan Ch, Bagavan A.Z., Abdul A., Elango G., Kamaraj Ch (2011). Evaluation of green synthesized silver nanoparticles against parasites.

[22] Vu X.H. (2018). Synthesis and study of silver nanoparticles for antibacterial activity against Escherichia coli and Staphylococcus aureus.

[23] Verma A., M.S.J.J.o.r.R. Mehata, and a. sciences (2016). Controllable synthesis of silver nanoparticles using Neem leaves and their antimicrobial activity.

[24] Rejiniemon T.S. (2014). n-vitro antimicrobial, antibiofilm, cytotoxic, antifeedant and larvicidal properties of novel quinone isolated from Aegle marmelos (Linn.) Correa. I.

[25] Sharma V.K. (2009). Silver nanoparticles: green synthesis and their antimicrobial activities.

[26] Bhattacharyya A. (2011). Disaster Risk Vulnerablity Conference.

[27] Shaik M.R. (2018). Plant-extract-assisted green synthesis of silver nanoparticles using Origanum vulgare L. extract and their microbicidal activities.

[28] Swilam N., Nematallah K.A.J.S.R. (2020). Polyphenols profile of pomegranate leaves and their role in green synthesis of silver nanoparticles.

[29] Selim Y.A. (2020). Green synthesis of zinc oxide nanoparticles using aqueous extract of Deverra tortuosa and their cytotoxic activities.

[30] Huq, M.A.J.I.j.o.m.s. (2020). Green synthesis of silver nanoparticles using Pseudoduganella eburnea MAHUQ-39 and their antimicrobial mechanisms investigation against drug resistant human pathogens.

[31] Ullah S. (2023). Advancing photocatalysis: Innovative approaches using novel V2O5/ZnO nanocomposites for efficient photocatalytic degradation of tubantin red.

[32] Mali S.C. (2020). Green synthesis of copper nanoparticles using Celastrus paniculatus Willd. leaf extract and their photocatalytic and antifungal properties.

[33] Sohal J.K. (2019). Determination of antioxidant potential of biochemically synthesized silver nanoparticles using Aloe vera gel extract.

[34] Anilakumar K. (2010).

[35] Niko N.J.J.o.B., World T.s. (2016). Inhibitory effects of Aloe vera gel aqueous extract and L. casei against E. coli in yoghurt.

[36] Salmaoui S., Sediri F., Gharbi N.J.P. (2010). Characterization of h-WO3 nanorods synthesized by hydrothermal process.

[37] Ghosh S. (2012).

[38] Golla, N.J.M.D.D.D.T. (2018). Phytosynthesis and antimicrobial studies of silver nano particles using Ziziphus nummularia leave extracts.

[39] Ajel M.K., Al-Nayili A.J.E.S., Research P. (2023). Synthesis, characterization of Ag-WO3/bentonite nanocomposites and their application in photocatalytic degradation of humic acid in water.

[40] Shahid W. (2022). Ex situ synthesis and characterizations of MoS2/WO3 heterostructures for efficient photocatalytic degradation of RhB.

[41] Matalkeh M. (2022). Visible light photocatalytic activity of Ag/WO3 nanoparticles and its antibacterial activity under ambient light and in the dark.

[42] Sungpanich J., Thongtem T., Thongtem S.J.J.o.N. (2014). Photocatalysis of WO3 nanoplates synthesized by conventional-hydrothermal and microwave-hydrothermal methods and of commercial WO3 nanorods.

[43] Salmaoui S. (2013).

[44] Mansur H.S. (2008). FTIR spectroscopy characterization of poly (vinyl alcohol) hydrogel with different hydrolysis degree and chemically crosslinked with glutaraldehyde.

[45] Logeswari P., Silambarasan S., J.J.J.o.S.C.S. Abraham (2015). Synthesis of silver nanoparticles using plants extract and analysis of their antimicrobial property.

[46] Nejatzadeh-Barandozi F., Enferadi S.T.J.O., Letters M.C. (2012). FT-IR study of the polysaccharides isolated from the skin juice, gel juice, and flower of Aloe vera tissues affected by fertilizer treatment.

[47] Vanaja M., Annadurai G.J.A.n. (2013). Coleus aromaticus leaf extract mediated synthesis of silver nanoparticles and its bactericidal activity.

[48] Najafi-Ashtiani H. (2018).

[49] Mir A., Shabani-Nooshabadi M.J.I.S.J. (2023).

[50] Parvatikar N. (2006). Electrical and humidity sensing properties of polyaniline/WO3 composites.

[51] Hiroshiba N. (2014). Simple synthesis conditions of WO3 hydrate nanorods by solvothermal process.

[52] Krašovec U.O., Vuk A.Š., Orel B.J.E.A. (2001). IR Spectroscopic studies of charged–discharged crystalline WO3 films.

[53] Zhang J. (2019).

[54] Wang F., Di Valentin C., G.J.T.J.o.P.C.C. Pacchioni (2011). Electronic and structural properties of WO3: a systematic hybrid DFT study.

[55] Tahir M.B. (2018). WO 3 nanostructures-based photocatalyst approach towards degradation of RhB dye.

[56] Polleux J. (2006). Template‐free synthesis and assembly of single‐crystalline tungsten oxide nanowires and their gas‐sensing properties.

[57] Renuka R. (2020).

[58] Biswal S.K. (2021). Green synthesis of silver nanoparticles using raw fruit extract of mimusops elengi and their antimicrobial study.

[59] Warsi A.-Z. (2022).

[60] Tahir M. (2018).

[61] Ahmed B. (2018). Well-controlled in-situ growth of 2D WO3 rectangular sheets on reduced graphene oxide with strong photocatalytic and antibacterial properties.

[62] Tijani J.O. (2019). One-step green synthesis of WO 3 nanoparticles using Spondias mombin aqueous extract: effect of solution pH and calcination temperature.

[63] Arunachalam R. (2012). Phytosynthesis of silver nanoparticles using Coccinia grandis leaf extract and its application in the photocatalytic degradation.

[64] Leaves L., Leaves L.J.A.J.o.E. (2014). Antioxidant activity by DPPH radical scavenging method of ageratum conyzoides.

[65] Covaliu C.I. (2010). Radical scavenger properties of oxide nanoparticles stabilized with biopolymer matrix.

[66] Koleva I.I. (2002). Screening of plant extracts for antioxidant activity: a comparative study on three testing methods.

[67] Gupta S.S. (2014).

[68] Paul S. (2009). Investigation of antioxidant property of iron oxide particlesby 1′-1′ diphenylpicryl-hydrazyle (DPPH) method.

[69] Uzair M. (2023). Effect of Mn Doped on Structural, Optical, and Dielectric Properties of BiFe1–x Mn x O3 for Efficient Antioxidant Activity.

[70] Yeo J., Shahidi F.J.J.o.F.B. (2019).

[71] Habeeb Rahuman H.B. (2022). Medicinal plants mediated the green synthesis of silver nanoparticles and their biomedical applications.

